# Point-Level Fusion and Channel Attention for 3D Object Detection in Autonomous Driving

**DOI:** 10.3390/s25041097

**Published:** 2025-02-12

**Authors:** Juntao Shen, Zheng Fang, Jin Huang

**Affiliations:** 1 School of Electrical Engineering, Southwest Jiaotong University, Chengdu 611756, China; yunyee@my.swjtu.edu.cn; 2 Sichuan Institute of Land Science and Technology (Sichuan Center of Satellite Application Technology), Chengdu 610072, China; fangzheng_sclst@163.com

**Keywords:** autonomous driving, 3D object detection, multimodal fusion, channel attention mechanism, ECA

## Abstract

As autonomous driving technology progresses, LiDAR-based 3D object detection has emerged as a fundamental element of environmental perception systems. PointPillars transforms point cloud data into a two-dimensional pseudo-image and employs a 2D CNN for efficient and precise detection. Nevertheless, this approach encounters two primary challenges: (1) the sparsity and disorganization of raw point clouds hinder the model’s capacity to capture local features, thus impacting detection accuracy; and (2) existing models struggle to detect small objects within complex environments, particularly regarding orientation estimation. To address these issues, we propose two enhancements: (1) point-level fusion of LiDAR point clouds and RGB images, which integrates the semantic information of 2D images with the geometric features of 3D point clouds to improve model performance in intricate scenarios; (2) the incorporation of the Efficient Channel Attention mechanism to concentrate on essential features, particularly for small and sparse objects. Experimental results on the KITTI dataset indicate significant improvements, particularly in small object detection tasks, such as identifying pedestrians and cyclists. The enhanced model also demonstrates substantial gains in the Average Orientation Similarity (AOS) metric. These enhancements enhance the vehicle’s ability to track and predict object trajectories in dynamic environments, critical for reliable recognition and decision-making.

## 1. Introduction

Autonomous driving technology is pivotal in enhancing road safety, efficiency, and convenience [1,2]. Environmental perception systems, fundamental components of autonomous driving, are engineered to perceive and interpret the surrounding environment in real time [3]. Central to these systems is 3D object detection technology, which facilitates the recognition and localization of objects, such as vehicles, pedestrians, and traffic signs, within a three-dimensional space. This capability is essential for the safe navigation of autonomous vehicles [4].

Traditional object detection methods predominantly rely on handcrafted features and rule-based algorithms, such as Haar-like features [5] and Histogram of Oriented Gradients (HOG) [6]. While these methods have shown success in controlled environments, their performance is limited in complex and dynamic traffic scenarios. The emergence of deep learning has revolutionized object detection, particularly with the introduction of convolutional neural networks (CNNs) [7], which automatically learn hierarchical features from large-scale image data. Notable algorithms, including R-CNN [8], YOLO [9], and SSD [10], have significantly advanced 2D object detection. However, these approaches encounter challenges in capturing the 3D spatial information that is critical for autonomous driving applications.

The integration of LiDAR sensors with monocular cameras has emerged as an effective solution for 3D object detection in autonomous vehicles [11]. LiDAR delivers precise spatial information through point cloud data, while monocular cameras capture rich color and texture details that LiDAR lacks. The combination of these complementary modalities has the potential to significantly enhance the accuracy and robustness of 3D object detection systems. However, in complex traffic scenarios—especially when detecting small or occluded objects—effectively fusing these data sources to achieve both real-time performance and high detection accuracy presents a significant challenge.

In recent years, several deep learning-based methods have been proposed to tackle the 3D object detection problem. PointNet [12] and PointPillars [13] are two prominent approaches that process point clouds directly. While these methods demonstrate promising results, they primarily concentrate on local or global feature extraction without fully leveraging attention mechanisms to dynamically emphasize relevant features during detection. Furthermore, existing algorithms often prioritize object detection accuracy at the expense of precision in orientation prediction.

There remain unresolved questions concerning the optimal strategies for incorporating attention mechanisms into 3D object detection frameworks, particularly within the context of multimodal data fusion [14]. Some researchers advocate for early fusion strategies, while others favor late fusion [15], highlighting the necessity for further exploration to optimize multimodal fusion techniques for 3D object detection.

This study introduces a novel multimodal 3D object detection framework that integrates a channel attention mechanism [16] with the PointPillars architecture. By combining the PointPainting [17] algorithm for multimodal data fusion with channel attention, we aim to enhance small object detection and improve the accuracy of orientation prediction. The primary contributions of this work are as follows:

1. We effectively leverage the complementary strengths of LiDAR and monocular image data as inputs to the multimodal 3D object detection model.

2. We incorporate a channel attention module [16] into the PointPillars network to dynamically emphasize relevant features, thereby enhancing feature extraction at both local and global scales.

3. Comprehensive experiments on the KITTI dataset [18] demonstrate that the proposed model surpasses existing methods in detecting small and occluded objects, resulting in significant improvements in orientation prediction accuracy

In conclusion, this study enhances vehicle perception through multimodal data fusion and attention-based feature selection, thereby contributing to the advancement of 3D object detection for autonomous driving. These improvements not only increase detection accuracy but also establish a more reliable foundation for real-time decision-making in autonomous systems.

## 2. Related Work

### 2.1. 3D Object Detection Methods

Existing 3D object detection models can be broadly classified into two categories: single-stage [19] and two-stage frameworks [20]. Single-stage networks directly predict the final 3D bounding boxes, whereas two-stage networks first generate candidate bounding boxes and subsequently refine them to produce the final 3D boxes. In terms of data processing approaches, contemporary 3D object detection algorithms are typically categorized into those based on raw point clouds [21], voxel- or pillar-based methods [22], point cloud and voxel fusion [23], and multi-view or multimodal approaches [24].

#### 2.1.1. Raw Point Clouds Methods

The raw point cloud-based method entails the direct utilization of point cloud data obtained from LiDAR for three-dimensional object detection and recognition, without converting it into alternative representations such as voxels or projections. This approach is advantageous due to its efficiency and straightforwardness. However, raw LiDAR point cloud data is inherently sparse and unordered, rendering it unsuitable for direct processing by neural networks. To address this challenge, Qi et al. introduced the innovative PointNet [12] algorithm for object detection in 2017. PointNet extracts features from raw point clouds through the application of T-Net transformation matrices and fully connected layers (multi-layer perceptron, MLP), followed by a max-pooling operation to derive global features. While PointNet effectively manages point cloud disorder, its focus on global feature extraction restricts its ability to capture local details, and the max-pooling operation may lead to the loss of valuable information. To mitigate these limitations, Qi et al. proposed PointNet++ [25], which employs a hierarchical approach.

#### 2.1.2. Voxel-Based Methods

Voxel-based methods partition space into uniform cubes (voxels) and allocate each point in the scene to a specific voxel. By extracting and learning features within each voxel, this approach streamlines the processing of point clouds. The voxelization technique was initially introduced by VoxelNet [26], which segments point cloud data into uniformly spaced voxels and utilizes a Voxel Feature Encoding (VFE) layer to transform the points within each voxel into feature vectors. This architecture enables the network to stack multiple VFE layers for the learning of complex feature representations. However, due to its reliance on computationally intensive 3D convolution operations, detection speed can be relatively slow. The SECOND [27] network mitigated this issue by integrating sparse convolutions as a replacement for traditional 3D convolutions.

#### 2.1.3. Image-Point Cloud Fusion

RGB-based 2D detection and 3D object detection utilizing point clouds each present distinct advantages while also facing specific limitations. Methods relying solely on images lack spatial depth information and are sensitive to variations in lighting, weather, and viewpoint. In contrast, approaches that depend exclusively on 3D point clouds encounter challenges such as low detection accuracy for small objects, high computational complexity due to large data volumes, and substantial costs associated with LiDAR and depth cameras. Therefore, integrating image and point cloud data for multimodal object detection is crucial for autonomous driving.

In 2017, Chen et al. proposed the MV3D [24] object detection network, which combines 2D image features, bird’s-eye view (BEV), and front-facing view data within the point cloud scene. In 2020, Sourabh Vora introduced the PointPainting [17] network, which employs a sequential fusion approach for multimodal object detection and has been widely adopted. In 2021, the 3DVG–Transformer [28] network, based on the Transformer architecture, utilized vision guidance to integrate multimodal data in a global context, effectively enhancing detection accuracy. In 2023, the Multi-Modal Fusion [29] algorithm processed multimodal data from various sensors during feature extraction through a deep learning model, integrating the multimodal information using a feature fusion layer.

### 2.2. PointPillars

**Figure 1:**
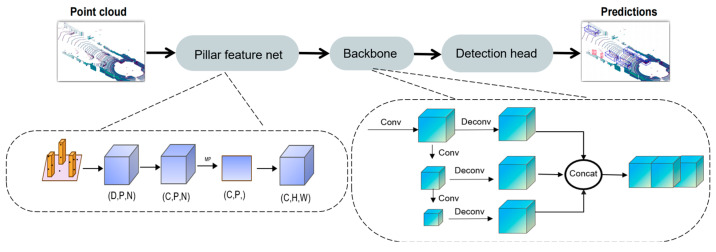
Architecture of PointPillars.

The PointPillars [13] algorithm efficiently conducts 3D object detection by converting point cloud data into bird’s-eye view (BEV) style 2D pseudo-images. It comprises three main components: the Pillar Feature Net (PFN) layer, which transforms point cloud data into pseudo-images; the Backbone network (a 2D CNN), which extracts spatial and semantic information among pillars; and the SSD [10] detection head, responsible for object classification and bounding box regression. By employing 2D convolutions instead of 3D convolutions, PointPillars [13] enhances computational efficiency and alleviates the limitations associated with fixed voxel sizes, particularly excelling in the detection of small objects, such as pedestrians and cyclists. However, the conversion of point clouds into pillars results in the loss of height dimension information, which may impair performance in complex scenes and reduce the accuracy of object orientation angle predictions, particularly when compared to algorithms that depend on fine-grained 3D convolutions. The architecture of PointPillars is shown in Figure 1.

## 3. Approach

In this section, we provide a detailed description of the proposed method. First, we present an overview of the 3D object detection architecture, followed by an explanation of the multimodal data fusion strategy. Finally, we discuss the incorporation of attention mechanisms along the channel dimension.

### 3.1. Overall Architecture

**Figure 2:**
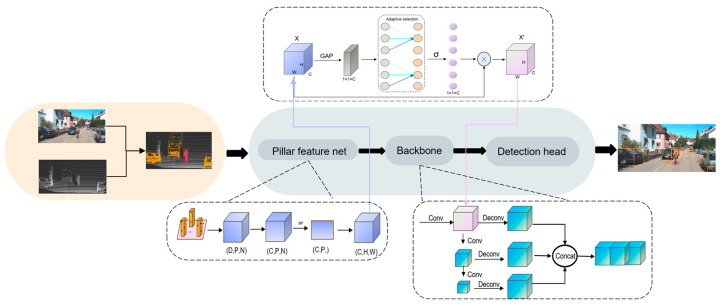
Overall architecture.

The overall structure of the proposed model is depicted in the accompanying Figure 2. Traditional 3D object detection models typically utilize LiDAR’s 3D point clouds as input. In contrast, we adopt a point-level fusion strategy known as PointPainting [17], which employs a 2D semantic segmentation network to extract the target object’s pixels from the RGB image. These pixels are subsequently projected onto the LiDAR point cloud to create enhanced point cloud data. The colored points are then input into the PointPillars [13] network to execute the 3D object detection task. Additionally, we integrate the Efficient Channel Attention (ECA) [16] net to compute local mutual relationships between channels in the Pillar Feature Net layer and the 2D CNN. The resulting data is applied to the network’s feature map, thereby augmenting the model’s detection capabilities.

### 3.2. Multi-Modal Data Fusion Strategy

The innovation of the PointPainting [17] algorithm lies in its capacity to effectively integrate image semantic information with point cloud spatial data, thereby providing more accurate and robust input for subsequent object detection and recognition tasks.

We adopt PointPainting [17] as the multimodal data fusion strategy, which utilizes both point clouds and images as inputs to estimate oriented 3D bounding boxes. The algorithm comprises three key stages: (1) Semantic segmentation: Compute pixel-wise segmentation scores using the image semantic segmentation network. (2) Fusion: Project the segmentation scores onto the LiDAR point cloud to effectively color it. (3) 3D object detection: Perform object detection using a LiDAR-based 3D detection network. PointPainting [17] integrates semantic information into the point cloud by projecting the point cloud onto the image plane, extracting corresponding image features, and subsequently “painting” these features back onto the point cloud data, resulting in an enhanced point cloud.

We utilize the KITTI dataset [18] for multimodal data fusion. Each LiDAR point in the KITTI dataset comprises four dimensions: (x, y, z, r), where (x, y, z) represent the 3D spatial coordinates and r denotes the reflection intensity. The semantic segmentation network outputs C class scores (s_0_, s_1_, s_2_, …, s_n_), where C = 4 (car, pedestrian, cyclist, background). Once the LiDAR points are projected onto the image plane, the semantic scores of the corresponding pixels (x_img, y_img) are appended to the LiDAR points, resulting in augmented LiDAR points in the form (x, y, z, r, s_0_, s_1_, s_2_, s_3_).

### 3.3. Channel Attention Module

**Figure 3:**
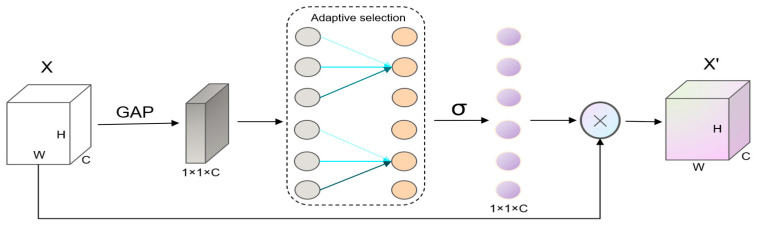
Efficient Channel Attention.

Convolutional neural networks (CNNs) typically assign equal weights to all features during feature extraction, which can result in the inclusion of many irrelevant features and an increase in computational cost. To address this issue and enhance the extraction of relevant features, we optimize the network structure by integrating attention mechanisms, thereby improving the algorithm’s performance in detecting small targets. The backbone of the PointPillars [13] algorithm employs a CNN for feature extraction. We incorporate the Efficient Channel Attention (ECA) mechanism [16] into the convolutional layers of the backbone, introducing attention computation along the channel dimension. This modification effectively directs the model’s focus toward critical features, enhancing detection accuracy and overall performance. Its architecture is shown in Figure 3.

The principle of the ECA mechanism [16] in CNN involves the use of a dimension-preserving global average pooling (GAP) to aggregate convolutional features, followed by an adaptive determination of kernel size k, one-dimensional convolution, and learning channel attention via the Sigmoid function to enhance the model’s performance in feature processing. The two core steps of ECA are global average pooling and one-dimensional convolution. The ECA module first applies global average pooling to the feature map of each channel, compressing the 2D feature map of each channel into a C × H × W format:

(1) $$z_{c}=\frac{1}{H\times W}\sum_{i=1}^{H}\sum_{j=1}^{W}X_{c}(i,j),c=1,2,\ldots ,C$$

Assume that the input feature map X has dimensions $X\in R^{C\times H\times W}$, where C represents the number of channels, and H and W denote the spatial resolution of the feature map. Each channel encodes specific information, such as edge features, color, and depth. The features of small objects are often concentrated in only a few channels, while other channels may contain background or irrelevant information. Global average pooling performs spatial compression on each channel, reducing the H×W feature to a single scalar value, yielding the global feature description z for each channel. Among them, $z_{c}$ is the average value of channel C, and finally a channel vector $z\in R^{C}$ is obtained.

One-dimensional convolution refers to the convolution operation performed along a single dimension. Its primary objective is to capture local interactions between channels, specifically the interdependencies among different channels. Let $y\in R^{C}$ denote the input channel feature vector, and the one-dimensional convolution operation can be expressed as follows:

(2) $$w=Conv1D(z),w\in R^{C}$$

The adaptive convolution kernel dynamically adjusts the kernel size, enabling each convolution operation to capture cross-channel interactions based on the number of channels (i.e., the dimensionality of the input features). This can be expressed by the following equation:

(3) $$k=\psi \left(C\right)={\left|\frac{\log_{2}\left(C\right)}{\gamma }+\frac{b}{\gamma }\right|}_{odd}$$

where

k: The output channel weight.

$\psi \left(C\right)$: The function of the ECA module used to calculate the channel weights.

$\log_{2}\left(C\right)$: The logarithm to the base 2, used to transform the number of channels into a logarithmic scale, which helps maintain smooth changes in weights when the number of channels varies.

γ: A scaling factor that adjusts the weight of the logarithmic scale.

b: A bias term that adjusts the offset of the weights.

${\left|\frac{\log_{2}\left(C\right)}{\gamma }+\frac{b}{\gamma }\right|}_{odd}$: Represents the operation of taking the odd part, which is a key feature of the ECA module. This ensures that the output weights are odd numbers. This is important because these weights are used to calculate the weights of each channel in subsequent processing. Odd weights can ensure a more uniform distribution of weights after the subsequent Sigmoid activation function, thereby improving the model’s expressive power.

Activate the convolution output weights $\omega_{i}$ using the Sigmoid function, where σ denotes the Sigmoid function. The Sigmoid function effectively squashes the output weights to a range between 0 and 1.

$$\alpha_{c}=\sigma (w_{c}),c\in \{1,2,\ldots ,C\}$$

Finally, the feature of each channel is multiplied by its corresponding weight, thereby adjusting the importance of each channel.

(4) $$\chi_{i}^{\prime }=\alpha_{c}\cdot \chi_{i}$$

## 4. Experimental Setup

In this section, we will provide a detailed description of the model framework we designed and the dataset details used.

### 4.1. KITTI and Evaluation Metrics

We used the KITTI dataset [18]. The KITTI official annotations categorize the dataset into three object classes: cars, pedestrians, and cyclists. It is also evaluated based on four detection metrics: BBOX, BEV, 3D, and AOS. BBOX refers to the accuracy of 2D bounding boxes; BEV indicates the accuracy of bounding boxes in the bird’s-eye view; 3D refers to the accuracy of 3D bounding boxes; and AOS measures the accuracy of the object’s rotation angle, which evaluates how accurately the system can determine the rotational direction of an object relative to a reference point. The difficulty of detection in the KITTI dataset is categorized based on factors such as whether the ground truth bounding box is occluded, the extent of occlusion, and the height of the bounding box. The specific categorization is shown in Table 1.

### 4.2. Implementation Details

**Architecture:** To enhance the accuracy of multimodal fusion between point clouds and images in the dataset, this paper employs the PointPainting [17] method. This approach maps semantic information from RGB images onto the point cloud, resulting in a “painted” point cloud that integrates semantic data. The painted point cloud is subsequently fed into the PointPillars [13] model for 3D object detection. Additionally, to improve the model’s feature extraction capabilities, a channel attention mechanism is introduced into the 2D CNN of PointPillars [13]. This mechanism adjusts the weights of different channels to enhance the model’s ability to capture essential information. Experimental results demonstrate that the PointPillars [13] model, augmented with channel attention, significantly outperforms competing models in detection accuracy, especially in the case of small object detection, on the KITTI dataset.

**Parameter details:** The KITTI dataset [18] contains 7481 training samples and 7518 test samples. According to [24], we split the 7481 training samples into 3712 for the training set and 3769 for the validation set. The voxelization scene range is (0,69.12) × (−39.68,39.68) × (−3,1), with voxel size (0.16,0.16,4). Each voxel can contain a maximum of 32-point clouds, with a maximum of 16,000 voxels processed during training and 40,000 during testing. The initial learning rate was 0.003. The learning rate increases during the first 40% of the training phase and gradually decreases during the remaining 60%. The classification loss weight was 1.0, the localization loss weight was 2.0, the direction loss weight was 0.2, and the box encoding weight was 1.0. The NMS threshold was set to 0.01, with a maximum of 500 boxes retained. The gradient clipping limit was set to 10 to prevent gradient explosion. The training set was trained for 80 epochs with a batch size of 4. The validation set was evaluated using the AP10 metric, with an IoU threshold of 0.7 for the Car class and 0.5 for both Pedestrian and Cyclist classes.

**Experimental Setup:** As shown in Table 2, we used the PointPillars [13] implementation from the open-source 3D object detection library OpenPCDet as the foundation for the program. The object detection network model was trained on a GPU, specifically an NVIDIA GeForce RTX 3090 with 32 GB of memory and 640 Tensor cores. The GPU uses CUDA version 11.7 The experiment’s CPU was an 11th Gen Intel^®^ Core™ i9-11900K @ 3.50 GHz with 16 cores, and the system had 128 GB of memory. The operating system used was Ubuntu 18.04.6, the deep learning environment was Anaconda 3, the framework was PyTorch 1.8.1, and the programming language was Python 3.9.12.

## 5. Results

We compared the PointPillars [13] algorithm, which integrates multimodal data, with attention modules of different types: spatial attention using the Squeeze-and-Excitation (SE) module [30], spatial + channel attention using the Convolutional Block Attention Module (CBAM) [31], and channel attention using our custom-developed Efficient Channel Attention (ECA) algorithm [16]. The overall performance of the method is represented using mean Average Precision (mAP) at three different difficulty levels: easy, moderate, and hard. This comparison allows us to assess the impact of each attention mechanism on the model’s ability to improve 3D object detection accuracy under varying conditions.

For easy, moderate, and difficult samples, the IoU threshold for the Car class is set to 0.7 for all levels, while the IoU threshold for other classes is 0.5. The detection categories are Car, Pedestrian, and Cyclist. We used the AP40 and AP10 evaluation metrics, which primarily highlight the advantages of our model in terms of the 3D and AOS metrics. We also tried different fusion methods such as a newer early fusion strategy the Multi-Modal Fusion [29] and a classic late fusion strategy MV3D (Multi-View 3D) [24], and show the comparison of AOS Metrics AP10 on the KITTI dataset.

As shown in the data from Table 3 and Table 4:

**Pedestrian Category:** Under the AP40 metric, our model outperforms all others in every scenario, achieving scores of 59.67%, 54.67%, and 50.03%, respectively. Similarly, under the AP10 metric, our model also surpasses all others in all scenarios, attaining scores of 59.69%, 55.94%, and 51.68%, respectively.

**Cyclist Category:** Under the AP40 metric, our model outperforms all others in every scenario, achieving scores of 82.49%, 61.43%, and 57.38%, respectively. Similarly, under the AP10 metric, our model also demonstrates superior performance in all scenarios, with results of 80.70%, 61.79%, and 58.14%, respectively.

As shown in the data from Table 5 and Table 6:

**Pedestrian Category:** Under the AP40 metric, our model outperforms all others in every scenario, achieving scores of 55.55%, 52.87%, and 50.32%, respectively. Similarly, under the AP10 metric, our model also surpasses all others in all scenarios, attaining scores of 54.95%, 52.69%, and 50.43%, respectively.

**Cyclist Category:** Under the AP40 metric, our model outperforms all others in every scenario, achieving scores of 90.92%, 73.70%, and 69.59%, respectively. Similarly, under the AP10 metric, our model also demonstrates superior performance in all scenarios, with results of 88.27%, 72.77%, and 68.93%, respectively.

The results are shown in the Table 7. We observed a significant decrease in prediction accuracy for Cyclist and Pedestrian, with no notable accuracy advantage for Car. Additionally, its higher computational complexity resulted in training times far exceeding those of our model.

### 5.1. Performance Analysis

For the Car category, the performance differences among all models in both AP10 and AP40 evaluations are minimal. This phenomenon can be attributed to the prominent geometric features of cars, which existing methods can effectively capture. Our model primarily enhances small object detection, resulting in a limited impact on the detection accuracy of large objects, such as cars. Conversely, for pedestrian and cyclist detection, the ECA attention mechanism exhibits significant effectiveness, particularly in the AOS metric, for the following reasons.

**Optimized Algorithm Combination:** PointPainting performs early fusion by integrating image features with LiDAR point cloud features prior to their input into the network. This mapping of semantic information from images to each point in the LiDAR point cloud enhances the representation of point cloud features. By supplementing geometric information from LiDAR with semantic information provided by images, it significantly improves the accuracy of 3D object detection, particularly in scenarios where point clouds are sparse or heavily occluded. Our primary network, PointPillars, is an efficient object detection method that segments LiDAR point cloud data into pillars and utilizes convolutional neural networks (CNNs) for feature extraction. Compared to traditional voxel methods, the pillar method retains spatial structure while significantly reducing computational overhead. Thanks to its lightweight pillar structure and convolutional network, PointPillars effectively reduces computation while achieving point-level sequential fusion of point clouds and images through PointPainting, all without increasing computational burden, thereby significantly enhancing detection accuracy.

Early fusion enables simultaneous consideration of image and point cloud information during feature extraction, which reduces the complexity of computation and information transfer typically associated with late fusion. This approach is well-suited for real-time autonomous driving applications, particularly for vehicles with limited hardware resources. The methods maintain strong performance even in embedded systems or resource-constrained environments.

Moreover, we contend that the 3D object detection task in autonomous driving inherently contains substantial information redundancy and noise. Thus, an effective mechanism is required to assist the model in selectively fusing information from different modalities (images and point clouds). The advantage of the Efficient Channel Attention (ECA) mechanism lies in its ability to dynamically adjust channel weights, allowing for improved focus on features that contain more informative content, particularly in complex scenarios and sparse point cloud conditions.

**Figure 4:**
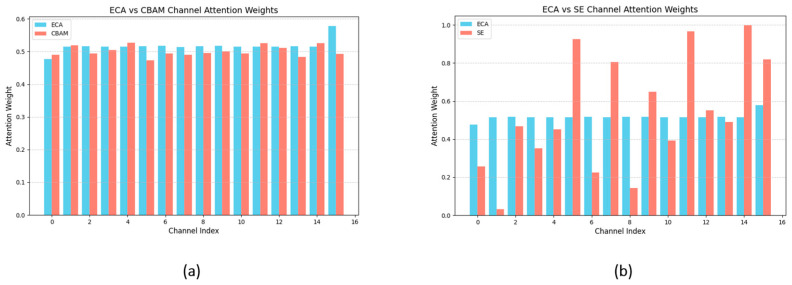
(**a**) ECA vs. CBAM channel attention weights; (**b**) ECA vs. SE channel attention weights.

**Efficient Attention Mechanism**: Small object detection typically encounters challenges related to sparse features and background interference. In such cases, attention mechanisms enhance the model’s feature selectivity, enabling it to automatically focus on the most important aspects of the input data. ECA adapts the convolution kernel size to capture inter-channel dependencies, resulting in a more uniform distribution of attention weights. This adaptation promotes a balanced consideration of the importance of different channels. Such a uniform distribution aids the model in avoiding the neglect of important channels while preventing excessive focus on certain channels, consequently enhancing the recognition accuracy of small targets. In contrast, the attention weight distributions of SE and CBAM are comparatively uneven, with some channels receiving disproportionately high weights while others retain relatively low weights. Furthermore, ECA is computationally efficient, yielding performance improvements for the model without significantly increasing the computational burden. The comparison results are shown in Figure 4.

### 5.2. Ablation Study

In this section, we conduct a comprehensive ablation study to validate the effectiveness of the proposed enhancements in our method. Painted PointPillars [13] served as our baseline model, being a widely adopted single-frame 3D object detection approach. Our ablation studies utilized four distinct evaluation metrics to assess the model’s performance from various perspectives: bounding box (bbox), bird’s-eye view (BEV), 3D, and Average Orientation Similarity (AOS). The model was trained for 80 epochs, with automated evaluation on the validation set using the AP40 metric. The Intersection-over-Union (IoU) thresholds for pedestrians and cyclists were both set to 0.5. These metrics offer a holistic evaluation of the model’s ability to accurately localize objects, maintain spatial consistency, and address orientation challenges, particularly in the context of small object detection and orientation accuracy.

As shown in the data from the Table 8, the improved model (Ours) significantly outperformed the baseline model (Painted PointPillars) in both pedestrian and cyclist detection tasks.

**Pedestrian:** The bbox metric shows improvements of +5.41, +5.55, and +5.41 for Easy, Moderate, and Hard difficulty levels, respectively, indicating a significant enhancement in boundary box localization. The largest improvement is seen in AOS, with increases of +9.55, +9.53, and +9.17 for Easy, Moderate, and Hard difficulty levels, respectively, demonstrating significant improvements in pedestrian direction prediction.

**Cyclist:** The bbox metric shows improvements of +3.89, +4.35, and +3.90 for Easy, Moderate, and Hard difficulty levels, respectively, also demonstrating significant progress. The improvement in mAPaos is equally notable, with increases of +6.00, +7.35, and +6.73 for Easy, Moderate, and Hard difficulty levels, respectively, indicating substantial improvements in cyclist direction prediction.

### 5.3. Qualitative Analysis

**Figure 5:**
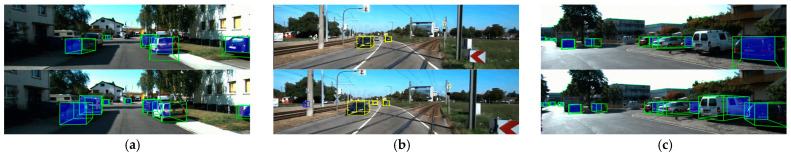
Quantitative analysis of three typical scenarios. (**a**) For occluded vehicles, our model has better performance than the original PointPillars model (**b**) For the small object on the left, our model has better performance than the original PointPillars model (**c**) highlights the improvement achieved by our model in detecting scenes where the target and environment exhibit low distinguishability.

As illustrated in Figure 5, we selected three distinct scenarios for qualitative analysis and comparison. In Figure 5a, the original PointPillars model exhibits suboptimal detection performance for overlapping and occluded targets, failing to detect the occluded white vehicle on the left. In contrast, our enhanced model demonstrates a significant improvement in performance. Figure 5b shows that the original model fails to recognize the small target on the left; however, our model successfully detects and frames the pedestrian with a blue bounding box. Lastly, Figure 5c highlights the substantial improvement achieved by our model in detecting scenes where the target and environment exhibit low distinguishability.

## 6. Conclusions and Future Work

### 6.1. Conclusions

In summary, the development of object detection technology for autonomous driving has reached a mature stage. Our team has identified that factors such as computational efficiency, cost, and the accuracy of small target detection play crucial roles in enhancing autonomous driving capabilities. While methods like PointPillars and PointPainting have found applications in multimodal data fusion, research on the integration of efficient attention mechanisms remains relatively sparse. We have innovatively introduced the Efficient Channel Attention (ECA) mechanism, a lightweight approach that minimizes redundant computations by optimizing the weighting of channels.

While other methods may improve performance by increasing model depth or complexity, we have prioritized low-cost and high-efficiency optimization strategies, emphasizing the practical benefits of our research and significantly enhancing the model’s generalization ability. Our focus is not solely on achieving the highest performance but rather on finding a balance between performance and computational efficiency. The ECA mechanism adapts weights dynamically, avoiding excessive computation on non-essential features, thus enabling the model to maintain low computational overhead while ensuring high precision, making it particularly suitable for the real-time demands of autonomous driving scenarios.

Although PointPillars, PointPainting, and ECA are established methods, we have compared various types of attention mechanisms and explored a range of multimodal fusion strategies through extensive experimental and theoretical validation. Ultimately, we have found this combination to be effective. Our research not only addresses real-time requirements, computational efficiency, and cost-effectiveness in autonomous driving but also enhances the detection accuracy of small targets. This makes our study practically applicable across a wide array of real-world scenarios.

Experimental results on the KITTI dataset [18] demonstrate that our method achieves significant improvements in small object detection compared to the baseline, particularly excelling in AOS and 3D AP scores across all difficulty levels. The findings validate that the combination of multimodal fusion and attention mechanisms effectively enhances detection performance, especially for small dynamic objects.

### 6.2. Future Work

Innovation in multimodal fusion technology:

1. Dynamic adaptive fusion strategy: In the future, we can explore dynamic fusion strategies to dynamically adjust the fusion weights of image and point cloud features according to the complexity of the input scene (such as point cloud density or lighting conditions), thereby improving fusion efficiency and detection performance.
2. End-to-end multimodal fusion: The current method uses an independent preprocessing step of PointPainting. In the future, we can try an end-to-end multimodal learning framework, such as directly jointly modeling images and point clouds through a shared feature extractor to reduce the time overhead of data preprocessing.
3. Robust fusion method: For the alignment error between point clouds and images in dynamic scenes, learning-based alignment methods can be introduced in the future, such as using image depth estimation or time series-based point cloud prediction methods to improve the robustness of inter-modal alignment.

Improvement of attention mechanism:

1. Joint attention mechanism: Based on ECA, explore the spatial channel joint attention mechanism and design attention mechanisms that can process different modalities simultaneously, such as the Transformer-based cross-modal attention mechanism, which directly captures the global interaction information between images and point clouds. This enables the model to focus on both spatial and channel information in the fused features, especially improving performance in the case of complex backgrounds or small target occlusions.
2. Lightweight global attention mechanism: In the future, a lightweight global attention mechanism optimized for 3D point clouds can be designed by combining the multi-level attention mechanism, so that the model can capture the global and local relationships between point clouds and image features without significantly increasing the computational cost, and further optimize the recognition and positioning capabilities of small targets.

## Figures and Tables

**Table 1:** KITTI dataset categorization.

Detection Difficulty	Minimum Bounding Box Height	Maximum Occlusion Level	Maximum Truncation Level
Easy	40	Fully Visible	15%
Moderate	25	Partially Occluded	30%
Hard	25	Difficult to See	50%

**Table 2:** Experimental configuration.

System	Version
CPU	11th Gen Intel^®^ Core™ i9-11900K @ 3.50GHz × 16
GPU	NVIDIA Geforce RTX 3090 Founders Edition
CUDA	11.7
Operating System	Ubuntu 18.04.6
Memory	64G
Deep Learning Environment	Anaconda 3
Deep Learning Framework	Pytorch 1.8.1
Programming Language	Python 3.9
Dataset	KITTI
Code Repository	OpenPCDet

**Table 3:** Comparison of 3D metrics AP40 on the KITTI dataset (%).

	Car			Pedestrian			Cyclist		
	Easy	Mod	Hard	Easy	Mod	Hard	Easy	Mod	Hard
PointPillars	87.39	78.24	75.36	57.55	51.25	46.77	78.99	56.46	52.83
PointPillars + SE	87.36	78.36	75.44	58.57	53.01	48.8	80.93	60.62	56.59
PointPillars + CBAM	87.49	78.34	75.47	58.14	52.25	47.62	82.23	54.96	51.75
**Ours**	**88.34**	**78.08**	**75.38**	**59.67**	**54.67**	**50.03**	**82.49**	**61.43**	**57.38**

**Table 4:** Comparison of 3D metrics AP10 on the KITTI dataset (%).

	Car			Pedestrian			Cyclist		
	Easy	Mod	Hard	Easy	Mod	Hard	Easy	Mod	Hard
PointPillars	86.79	77.11	75.51	57.76	52.18	48.34	78.09	56.95	53.31
PointPillars + SE	86.22	77.10	75.48	58.6	54.32	49.79	79.25	61.15	57.37
PointPillars + CBAM	86.77	77.14	75.51	58.78	53.97	48.95	80.10	55.63	53.19
**Ours**	**86.76**	**76.86**	**75.60**	**59.69**	**55.94**	**51.68**	**80.70**	**61.79**	**58.14**

**Table 5:** Comparison of AOS metrics AP40 on the KITTI dataset (%).

	Car			Pedestrian			Cyclist		
	Easy	Mod	Hard	Easy	Mod	Hard	Easy	Mod	Hard
PointPillars	95.59	93.51	91.18	45.58	43.34	41.17	86.97	66.35	62.88
PointPillars + SE	95.48	92.18	91.26	47.68	46.05	44.07	89.16	75.04	70.96
PointPillars + CBAM	95.76	92.41	91.36	45.18	43.14	40.57	89.61	70.10	66.52
Ours	95.45	93.13	91.21	55.55	52.87	50.32	90.92	73.70	69.59

**Table 6:** Comparison of AOS metrics AP10 on the KITTI dataset (%).

	Car			Pedestrian			Cyclist		
	Easy	Mod	Hard	Easy	Mod	Hard	Easy	Mod	Hard
PointPillars	90.72	89.66	88.57	47.31	45.84	43.73	83.67	66.01	63.00
PointPillars + SE	90.71	89.60	88.59	46.91	45.93	44.04	85.32	73.99	70.36
PointPillars + CBAM	90.78	89.85	88.60	47.92	46.24	43.59	85.67	69.21	66.17
Ours	90.67	89.51	88.58	54.95	52.69	50.43	88.27	72.77	68.93

**Table 7:** Comparison of AOS metrics AP10 for different fusion strategies.

	Car			Pedestrian			Cyclist		
	Easy	Mod	Hard	Easy	Mod	Hard	Easy	Mod	Hard
MV3D	94.72	88.85	88.60	44.43	43.24	40.59	80.67	65.38	57.17
Multi-Modal Fusion	85.23	83.62	82.19	39.94	38.26	37.66	58.88	57.42	56.69
PointPillars	90.72	89.66	88.57	47.31	45.84	43.73	83.67	66.01	63.00
Ours	90.67	89.51	88.58	54.95	52.69	50.43	88.27	72.77	68.93

**Table 8:** Comparison of effectiveness of different evaluation indicators in KITTI dataset.

		PointPillars				Ours				
		Easy	Mod	Hard	mAP	Easy	Mod	Hard	mAP	Delta
Pedestrian	mAP_bbox_	70.28	66.13	63.37	**66.59**	75.56	71.68	68.78	**72.01**	**+5.41**
	mAP_bev_	63.96	57.30	53.59	**58.28**	64.91	59.89	55.80	**60.20**	**+1.92**
	mAP_3d_	57.55	51.25	46.77	**51.86**	59.67	54.67	50.03	**54.79**	**+2.93**
	mAP_aos_	45.58	43.34	41.17	**43.36**	55.55	52.87	50.32	**52.91**	**+9.55**
Cyclist	mAP_bbox_	88.59	71.54	67.83	**75.99**	92.00	75.89	71.73	**79.87**	**+3.89**
	mAP_bev_	86.06	63.15	59.08	**69.43**	87.35	66.95	62.51	**72.27**	**+2.84**
	mAP_3d_	78.99	56.46	52.83	**62.76**	82.49	61.43	57.39	**67.10**	**+4.34**
	mAP_aos_	86.97	66.35	62.88	**72.07**	90.92	73.70	69.59	**78.07**	**+6.00**

## Data Availability

KITTI is a project of Karlsruhe Institute of Technology and Toyota Technological Institute at Chicago. It is available at https://www.cvlibs.net/datasets/kitti/ (accessed on 13 May 2024) with permission.
